# A Proposal for a National Cancer Control Plan for the UAE: 2022–2026

**DOI:** 10.3390/clinpract12010016

**Published:** 2022-02-17

**Authors:** Humaid O. Al-Shamsi, Amin M. Abyad, Saeed Rafii

**Affiliations:** 1 Department of Oncology, Burjeel Cancer Institute, Burjeel Medical City, Abu Dhabi P.O. Box 92510, United Arab Emirates; amin.abyad@burjeelmedicalcity.com; 2 Innovation and Research Center, Burjeel Cancer Institute, Burjeel Medical City, Abu Dhabi P.O. Box 92510, United Arab Emirates; 3 College of Medicine, University of Sharjah, Sharjah P.O. Box 27272, United Arab Emirates; 4 Emirates Oncology Society, Dubai P.O. Box 6600, United Arab Emirates; s.rafii@gmail.com; 5 Department of Oncology, Saudi German Hospital, Dubai P.O. Box 391093, United Arab Emirates

**Keywords:** cancer control, prevention, middle east, arab, registry

## Abstract

The United Arab Emirates (UAE) is one of the fastest growing economies with consequent increase in non-communicable diseases including cancer. The number of reported cases and mortality have been increasing in the UAE over the years, despite screening and early detection efforts which appear to be far from target coverage of the intended population. In this work, we highlight key elements of a proposed national cancer control plan for the UAE. The plan is still a work in progress and has not yet been officially adopted. A comprehensive and effective control plan requires accurate data, a reliable cancer registry, and periodic monitoring and evaluation. The UAE cancer control plan is being prepared in line with the WHO and EMRO framework, with defined objectives and goals. The objectives are to combat cancer, reduce incidence, control mortality, and improve outcomes and quality of life for cancer patients. There is also a focus on improving public health education, prevention, early detection, prompt diagnosis, treatment facilitation, continuity of care, performance evaluation, training of workforce, and research.

## 1. Introduction

Cancer is a major health problem globally, and the second leading cause of death in the world, with a mortality rate of 8.8 million people in 2015, accounting for approximately 1 out of 6 deaths [1]. Cancer is the fourth leading cause of death in the Eastern Mediterranean region, after cardiovascular diseases, infectious diseases, and trauma [2]. The rate of cancer appears to be increasing in the Middle East, mostly related to rapid population growth, increasing life expectancy, and urbanization with progressively westernized lifestyles [3]. The incidence of cancer is predicted to double in the next two decades, the highest expected increase among all WHO regions. It is expected that by 2030, cancer related deaths will reach 652,097 from 367,441 reported in 2012 [4]. These projections are based on the impact of population growth and aging, the additive effect of increased exposure to cancer risk factors, such as smoking, diet, physical inactivity, and environmental pollution, which would lead to a greater rise in cancer burden. This employs an enormous burden on healthcare services.

Cancer is the third leading cause of death in the United Arab Emirates (UAE) after cardiovascular diseases and trauma. In the UAE, the mortality-to-incidence ratio is 0.39, which is comparable to most developed countries (approximately 0.38). The crude cancer death rate is 10.26/100,000 people, with breast cancer ranking first (110 deaths), while colon cancer ranking second (98 deaths), and lung cancer ranking third (80 deaths) [5,6,7].

Cancer is a preventable disease in a significant number of cases. A substantial number of cancers can be detected in early stages and treated successfully. Even if diagnosed in late stages, cancer pain can be decreased, the disease progression slowed down, quality of life improved, and patients and their families facilitated to cope. Effective cancer control plans are, therefore, indispensable everywhere. In most countries, irrespective of economic status, some level of cancer control activity is ongoing. In many countries, there is no effective cancer control plan, the plan is out of date, or is not effectively implemented. There is thus a need for a comprehensive plan, its effective implementation, regular monitoring, quality assurance, and updating as per local needs and requirement [1,2,4].

Cancer, today, is managed by a multidisciplinary integrated approach with equity and uniform accessibility. The UAE aims to reduce the burden of cancer in the UAE and to make all possible efforts to deliver optimal cancer care for cancer patients and their families. It also aims to expand cancer care services in the UAE in line with international guidelines and the best global practices, adjusted to suit the needs of the country [3,6,7].

In this work we highlight key elements of a proposed national cancer control plan for the UAE. The plan is still work in progress and has not been yet officially adopted.

## 2. Cancer Burden in the UAE

During the mid-twentieth century, the UAE rose to become one of the fastest growing economies globally [8,9]. This growth leads to economic, sociodemographic, and lifestyle changes of the population, paralleled by an epidemiological increase in the rates of non-communicable diseases (NCD) and cancer. Subsequent studies in the UAE linked this increase in cancer with an exposure to several risk factors, including physical inactivity and sedentary lifestyles [10,11,12], the consumption of high-caloric and poorly nutritive meals [13], an increase in obesity [12,13], an increase in smoking [14], and elevated air pollutant levels [15]. There is an impact of improved cancer diagnostics. The reported deaths due to NCD in 2016 were 77.3% of all deaths. The main causes of deaths are cardiovascular diseases (40%) and cancer (12%). Communicable, maternal, perinatal, and nutritional conditions cause around 6% of all deaths, while chronic respiratory diseases and diabetes represent about 5% each [9,11].

### 2.1. Cancer Incidence Rate

A total of 4299 cases were diagnosed in 2017 in the UAE, including 4123 (95.91%) invasive cancer cases and 176 (4.09%) in situ cases. There were 2370 (55.1%) females and 1929 (44.9%) male. The cancer cases reported in 2016 were 4210 indicating a rise in 2017 from 2016, as per data of the annual reports of the National Cancer Registry in the United Arab Emirates. Invasive cancer cases are distributed according to the nationality in 2017, with 680 (62%) female cases and 425 (38%) male cases among UAE citizens, and 1570 (52%) female cases and 1448 (48%) male cases in non-UAE citizens [3,5,6].

### 2.2. The Most Common Cancer Types

The frequency of cancers in males and females as reported from the UAE in 2017 are summarized in Table 1. Breast is the most common site of cancer among the population of the UAE, accounting for 20.23% of all cancer cases in the year 2017. After breast cancer, colorectal cancer (10.24%), thyroid cancer (9.99%), leukemia (7.62%), skin (5.04%), non-Hodgkin’s lymphoma (4.17%), and prostate cancer (3.76%) are the most common types of cancers in UAE [3,6].

Table 2 shows the most common pediatric cancers in the UAE. Leukemia was the most common cancer among children in the UAE in 2017, accounting for 41.8% of all cancer cases in children [3,5,6].

### 2.3. Cancer Mortality

In 2017, cancer-related deaths in the UAE reached 955, about 10.82% of total deaths. The crude cancer death rate was 10.26/100,000 people, with breast cancer ranked first (110 deaths), while colon cancer ranked second (98 deaths), lung cancer ranked third (80 deaths), and leukemia ranked fourth (52 cases). The number of cancer deaths shows that the percentage of cancer deaths increased during the year per one thousand of the population in 2018 compared to 2017, which was adopted as a baseline for the index targets [3,6,7].

### 2.4. Cancer Prevention, Screening, and Early Detection

Around one third of cancers can be preventable by changes in lifestyle, genetic testing of high-risk groups, and vaccination. Even though cancer incidence is relatively low in the UAE, we must focus on improving the outcome of patients being treated for cancer. Nothing impacts cancer survival rates more than screening and early detection. Early detection of cancer is known to increase the chance of a successful treatment and improved outcome. Results for 2017 show a lower rate of population coverage for early cancer screening among the target age groups [<70%] than the targets set nationally or by the World Health Organization (Table 3) [1,2,4,5,16].

The causes of low screening rates in the UAE population and some actions recommended/proposed to improve these rates are described in Table 4 [5,6].

### 2.5. Diagnostic Services

A swift and definitive diagnosis is the key to prompt and definite treatment. We lack efficient and well-developed pathways to ensure that patients are referred to an appropriate physician and centers in time to be diagnosed within a minimum possible time. During this timeframe, patients are expected to complete a clinical physical assessment, radiologic imaging, and tissue diagnosis by biopsy and pathology. The plan for further assessment or management must be discussed and approved at multi-disciplinary team (MDT) tumor board meetings. All diagnostic services are not available universally and with the desired equity. We need tumor site-specific diagnostic clinics with a facility to support genetic testing. We will have to ensure quality assurance with international standards and quality control regulations, often requiring accreditation with recognized international organizations. This should come with regulation, development, and implementation of established diagnostic standards [5,17,18,19,20,21].

### 2.6. Cancer Management

It is a must to establish, improve, sustain, and monitor all-inclusive cancer services, including surgical oncology, medical oncology, radiation oncology, pediatric oncology, and palliative care services. There are newer sub-specialties evolving as gynecologic oncology, uro-oncology, osteo-oncology, neuro-oncology, among others. There is forthcoming necessity and demand to develop these specialized cancer care services with excellence, which are not up to the mark at present, nor in a true construct. We lack well-organized supportive services available to each and every patient such as clinical psychologist, clinical dietitians, social workers, and community nursing services at all levels of care provision. We are still far-away from perfection to develop and implement practice guidelines for referring physicians, radiologists, and pathologists for each cancer. These need to be formulated as per our local data, our set objectives and settings, local disease biology, and access to resources. It is essential to strengthen the role of the MDT tumor boards in cancer institutes and all new cases as well as complex cases must be reviewed and discussed by a MDT, to arrive at a well-documented plan of care for each individual [17,18,19,20,21]. Palliative care services are only available in comprehensive care centers.

### 2.7. Cancer Research

Overall, cancer research productivity is evolving over time, although several evidence gaps continue to persist. The scope for basic and translational research is generally expanding within the country with the continued rise in the number academic institutions and research programs dedicated to molecular and cellular research. Observational studies have been mostly focused on screening and basic parameters of epidemiology. Future directions should focus on expansion of national registries and longitudinal collection of clinically relevant variables that can better inform the clinical and molecular profiles of breast cancer in the country, and, more importantly, survival metrics that can help inform management strategies. Lastly, there is an evident paucity of therapeutic clinical trials, which is a challenge shared by the wider region. Several efforts are currently ongoing to solidify partnerships with clinical trial sponsors to host interventional studies in the country, considering the regulatory and resource infrastructures are well advanced and adaptable to international standards and requirements. There has to also be an equal effort placed on understanding modifiable lifestyle risk factors for cancer, such as smoking, obesity, diet, and physical inactivity, which are perceived to be in ‘bad shape’ in the UAE although true prevalence rates in Emiratis vs. expats and associations with cancer incidence remain unclear.

## 3. The UAE Cancer Control Plan

In 2011 [11,22,23], the WHO proclaimed the crucial demand to create a baseline for monitoring trends and to assess the progress of countries to contest the global epidemic of cancer in confronting this scourge. A cancer control plan needs accurate data assimilation, a consistent and progressive cancer registry, and monitoring and appraisal initiatives to ensure appropriate priority settings and to ensure quality [24]. The WHO advised governments to consolidate their health information systems with reliable and evidence-based research indicators.

The Ministry of Health and Prevention with its partners in the UAE started developing a national cancer control plan to be implemented in accordance with specific objectives. The plan, presented herein, is still a work in progress and has not been officially adopted. The proposed plan covers the years 2022–2026 and was prepared focused on the global action plan for prevention and control of cancer by the WHO and the regional executive framework for prevention and control of cancer for the Eastern Mediterranean Region (EMRO). The outline of this plan reflects the directions and strategy to reduce the cancer incidence and facilitate journey of cancer patients through their disease course, decrease mortality by at least 25%, and improve the quality of life through an integrated approach based on comprehensive activities and cancer care services. The plan is based on nine strategic axes centered across the healthcare system, services, prevention, sustainability, and innovation. It includes areas of governance, prevention, early examination, treatment, palliative care, surveillance, and research [2,6,16].

Development of the plan was made through a committee of local oncology practitioners and policy makers in co-operation with various stakeholders including both public and private sectors involved in clinical care and public health and education; to enrich commitment to global and regional initiatives to fight and control cancer. The plan is a strategic and general framework for the determinants and controls used in formulating policies, developing legislation, and assessing material resources to support the prevention, care, and management programs in the UAE. It further proposed appropriate measures for implementing the plan, continuous monitoring, and a follow-up system. Measurement indicators to evaluate the efficacy of suggested and adopted activities were also established. It represents the roadmap for the implementation of specifics of the plan for the prevention and control of cancer. This plan is aligned with the National Agenda of the UAE for 2021 and aims to reduce cancer death rates which will help achieve the objectives of the agenda [11,21,23,24]. The vision, objectives and strategy of the proposed UAE cancer control plan are summarized in Table 5.

### 3.1. The Guiding Principles and Strategic Directions of the UAE Cancer Control Plan as per WHO and EMRO Guidelines

The national cancer control plan is based on the strategic directions set by the executive framework of the action plan as pronounced by the World Health Organization for the Eastern Mediterranean Region. The recommendations of WHO and EMRO are summarized in Table 6 [4,11,16,24].

### 3.2. Definitions of the Strategic Axes and Executive Framework of the UAE Cancer Control Plan

The UAE cancer control plan should include 3 pivotal focus areas concentrated around nine strategic axes (Table 7).

The first is prevention, which revolves around the axes of education, understanding, prevention and detection, early and rapid diagnosis. The second focused area is that of healthcare and services which includes the axes of treatment and continuous care. The third area is that of sustainability and innovation and revolves around the axes of performance measurement, workforce, and research. These are further detailed and explained in depth in Table 8.

## 4. Vistas in the Cancer Control Plan

### 4.1. Expected Cancer Burden

The reported age-standardized cancer incidence in the UAE is low compared to the United States and other developed countries. There is, however, a real expectation to have an increment of cancer cases by 10–15% annually, keeping in sight existing cancer trends [2,3,5,6,7,8,11].

### 4.2. Cancer Control Program

An all-inclusive Cancer Control Program is mandatory to formulate strategy, implement prevention, and enforce a comprehensive cancer management through a collaborative process between government organizations, community, and non-government organizations (NGO). An effective and well-balanced plan should steer to risk reduction, early detection, and improved management to enhance survivorship and efficiently reduce the cancer burden.

The Cancer Control Program should be aimed at keeping the incidence rate at its current level or reducing it. A comprehensive national cancer program assesses various ways to control disease and implements most cost-effective and beneficial means for the population. It places emphasis on preventing cancers or detecting cases early at a curable stage and providing maximum possible relief to patients with advanced disease. A well-developed and implemented cancer control program should result in significant risk reduction, increasing early detection, improved treatment, and enhanced survivorship.

We must strengthen the National Cancer Control Committee, with members of the committee representing all stakeholders. We should adhere to the WHO Cancer Control Strategy to have a standard, reproducible, equitable and uniform approach. It is highly desirable to have increased collaboration with other ministries, such as the Ministry of Religious Affairs, Ministry of Education, and media outlets both private and public. We must draw a road map for the steps of adaptation of the WHO strategy based on a local database [8,11,22,23,24].

### 4.3. Cancer Registry

A cancer registry is the key instrument for any cancer control plan. It can define cancer burden, identify risk factors of cancer, and evaluate the cancer control program itself. A cancer registry can help in cancer control planning and organization through understanding cancer patterns and time trends by gathering epidemiological indicators, such as cancer incidence, mortality, prevalence, stage at diagnosis, and patient survival.

Recently, registry managers from 19 MENA countries reported the presence of 97 population-based, 48 hospital-based, and 24 pathology-based registries. Most population-based registries were well or partially developed. Lack of accurate death records, complete medical records, and communication between stakeholders and deficiencies in trained personnel were critical challenges that were more severe in active conflict zones and neighboring conflict-affected regions. Cancer registration challenges included weak health infrastructure, absence of legislation mandating cancer registration, and disruption of cancer registration because of active conflict and loss of funding [25].

A well-established national cancer registry regularly documents a yearly report on cancer incidence. Cancer registry should collect comprehensive information, such as stage of disease, mortality, and risk factor data. The registry often operates with an insufficient workforce, unavailability of data on patients diagnosed and treated abroad, and noncompliance from non-governmental institutes and medical personnel, and mobility of population [5,6,25,26,27]. To have precise, consistent, dependable, comprehensive, and beneficial cancer registry data, we need to

1. Establish the Cancer Registry Advisory Committee to participate in the planning of cancer registry activities, provide feedback on the data and reports, and contribute to data dissemination and advocacy for the registry;
2. Establishing and implementing data quality indicators;
3. To complete data on cancer stage, mortality, risk factors, and cancer prevalence;
4. Increase the trained human resources; work on retention of current staff through training more registry staff and to give them technical and financial support to ensure completeness of cancer registration and sustainability of the cancer registry;
5. Collect more inclusive data on cancers as well as non-morphologically;
6. Mandatory reporting, by working on legal obligation to notify cancer;
7. Adjustments of cancer notification form and electronic notification forms;
8. Seek more collaboration from healthcare providers.

### 4.4. Indicators for Monitoring

There are many known global Indicators of attainment employed for monitoring and progress of any national cancer control plan. There can be KPIs, with some being well known and some can be developed according to local situations. Examples of improvement include: education, measured by the cancer awareness measure; reduction in the numbers of smokers; increased proportion of stage 1 and 2 breast and colon cancer; increased proportion of patients diagnosed by screening or urgent referral pathways; publication and comparison of 1- and 5-year cancer survival rates; increase in patients with complete care plans in place across the care pathway; decreasing number of patients travelling abroad for treatment; and more patients accessing ongoing care in primary care/community care services [3,4,5,10,11,16,21,24].

### 4.5. Continuing the Path to Progress and Excellence

Certain progress has been made, but a lot more is needed as the vision for the future of cancer care remains unchanged as an enormous challenge. The UAE will continue to pursue excellence in cancer care by developing complex specialty services and innovative treatments. New, evidence-based approved treatment modalities and technologies, with proven impacts on patient care, will be accessed and incorporated in management strategies. Improvements in the patient experience will be achieved with a focus on the whole pathway of care, which is based upon the WHO Cancer Care Continuum [22,24,25,27].

### 4.6. Public Education and Understanding

It is an implacable requisite to invest in educating people to understand the importance of prevention, enhance prevention and early detection initiatives, and swift definite diagnosis of cancer. There is a need to improve refutation programs and alleviate myths about cancer. There are myths, such as that cancer appears at specific sites, appears only in late stages, is more common in low-income countries, is always fatal, and that surgery makes it spread quickly. There are multiple stigmas about cancer; people feel uncomfortable discussing cancer, avoid using the word cancer, diagnosis indicates weakness, fear of loss of job, and loss of sexual activity [2,4,26,27].

### 4.7. Partnership within the Cancer Community

There is a need to develop close associations within the cancer patient’s community with the objectives for enhancing awareness and prevention of cancer, understanding of cancer, sharing experiences, financial support for eligible cancer patients, to coordinate with various stakeholders to organize meetings, seminars, courses, and conferences; and to support research and raising awareness of the disease and its prevention [25,27].

## 5. Conclusions

The UAE is a growing economy with a substantial burden of cancer, which is expected to increase with consequent morbidity and mortality. There are screening and early detection efforts which are far from target coverage of the intended population. A comprehensive and effective national cancer control plan is always needed to effectively combat cancer. It needs accurate data collection, an efficient and organized cancer registry, and regular monitoring and evaluation of related activities. A UAE cancer control plan was herein proposed, in line with the WHO and EMRO cancer control initiatives and framework, with well-defined objectives and targets. The objectives are to combat cancer, decrease cancer incidence, restrict morbidity and mortality, improve outcomes, and augment the quality of life for cancer patients. In particular, there is a need to continue on the path of progress and excellence. The cancer registry needs to be broadened and given necessary legislative cover. We must introduce our efforts and commitment more in preventive oncology. We have to also incorporate the ever-growing knowledge, ongoing technological development, and newly approved medications as per international data and guidelines based on clinical trials. We should also endeavor to develop clinical pathways and guidelines, employ these, and continuously monitor our cancer services. We need to expand cancer care initiatives with participation of all stakeholders and deliver cancer services with equity and affordability, keeping in mind the issue of cost-effectiveness. There is a need to induct a qualified workforce, improve their skills and expertise by training and continuing education, and monitor their performance. It is essential to prepare and get international accreditations by known world organizations to be continuously on the path of progress.

The cancer care services must also be accessible with equity, reproducibility, and affordability. Appropriate distribution of resources can enhance delivery of cancer care, often at the patient’s doorstep. We need to integrate and link primary healthcare, secondary care hospitals, tertiary care, and private cancer care centers. The focus should be on development and investing in preventive oncology by boosting education, screening and early detection. The pathways of the patient journey from symptoms to definite treatment need to be thoroughly worked in line with local realities and international guidelines and protocols.

## Figures and Tables

**Table 1:** The most common primary malignant tumors in UAE for both genders, 2017 [3,5,6].

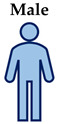		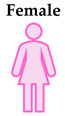	
Cancer Type	No. of Cases and %	Cancer Type	No. of Cases and %
Colorectal	256 (13.67%)	Breast	825 (36.67%)
Leukemia	196 (10.46%)	Thyroid	302 (13.42%)
Prostate	155 (8.28%)	Colorectal	166 (7.38%)
Skin	134 (7.15%)	Leukemia	118 (5.24%)
Lip, Oral cavity and Pharynx	112 (5.98%)	Uterus	111 (4.93%)
Thyroid	110 (5.87%)	Cervix uteri	82 (3.64%)
Non-Hodgkin Lymphoma	107 (5.71%)	Skin	74 (3.29%)
Bronchus and Lung	103 (5.50%)	Ovary	70 (3.11%)
Urinary bladder	91 (4.86%)	Non-Hodgkin Lymphoma	65 (2.89%)
Stomach	59 (3.15%)	Lip, Oral cavity, and Pharynx	39 (1.73%)

**Table 2:** Five commonest pediatric tumors in the UAE, 2017 [3,5,6].

Cancer Type	No. of Cases and %
Leukemia	61 (41.8%)
Kidney and Renal pelvis	11 (7.5%)
Non-Hodgkin Lymphoma	10 (6.8%)
Liver and intrahepatic bile ducts	8 (5.5%)
Brain and CNS	7 (4.8%)

**Table 3:** Screening targets and performance compared to the targets set nationally or by the World Health Organization (WHO)**,** based on unpublished data from the Ministry of Health and Prevention.

Type of Cancer	Target Age Group	Examination	% Of the Population Eligible for Screening Target Age Group	2017 Investigated Result	Population Coverage Ratio for Target Groups
Breast	Female ≥ 40 years	Mammogram every two years	50%	20,752	6.68%
Colon	Females and males ≥ 40–75 years	FIT Test after every 2 years	50%	18,399	1.6%
Cervix	Females All sexually active females, symptom free, aged 25–65 years	Cervical pap smear test should be after every 3 years	33%	39,281	7%

**Table 4:** Causes and reformatory actions to improve screening rates in the UAE.

Cause/Factor	Root Causes	Reformatory Actions
Access to the service	Difficulty in access to service Cost The location of examination centers Health insurance does not include screening Population growth and change in the population pyramid Younger population not in recommendation or guidelines	Mass education/campaign on screening and early detection Address the target groups at educational institutes, media, etc. Launch mobile screening services, such as Mammogram, Cervical Examination, Stool Examination (FIT test) medical and advice Covering the cost through health insurance, or delivering it free Develop discounted rates for screening packages Setting unified cost and quality for cancer screening Establish specialized center for cancer detection
Mechanism and quality control of cancer screening services	The dedication and commitment of health authorities and political leadership to the targets and indicators of screening and early detection programs The need of a unified national program for cancer screening Non-existent cancer screening programs Poor compliance in application of the national screening guidelines The absence of a dedicated team to monitor the quality of services Lack of human resources for auditing, and lack of audit to assess quality of services	Activating a national program for early detection of cancer that includes a central call system and text messaging to call the target high risk groups Establishing a mechanism and unified targets to measure the coverage rate Linking the Emirates ID to cancer screening record Committing all service providers in achieving the target percentage Activating the national registry for screening and early detection Integrating primary care and screening Building capacity and logistical resources Assigning coordinators for quality assurance Establishing screening services Establishing a Quality Assurance and monitoring department for screening services Integrating screening and early detection in insurance coverage Monitoring impact of screening on outcome mortality
Community awareness for the importance of early examination and Detection of Cancer	Lack of awareness campaigns Lack of education and understanding of cancer Lack of integration of information about cancer into the educational curricula Lack of availability of smart awareness applications Lack of awareness about early detection programs Lack of awareness of global awareness days for different types of cancer	Conducting a Cancer Awareness Measurement assessment by questionnaire in the community Intensifying awareness campaigns on the importance of early detection Information about the availability and locations of services Involving prominent and famous figures to disseminate awareness Emphasize the importance of early detection of cancer Develop a public information website Incorporating cancer information into educational programs, and campaigns in universities/schools Include representatives from the relevant authorities Conducting free campaigns to detect cancer during ambulatory care Smart awareness campaigns and applications to help community members make the right decisions and get early detection tests
End User factors influencing the screening and detection service	Emotional factors Fear Shyness Anxiety about the outcome Hospital Admission Practical factors Absence from Work Transportation Understanding and education Lack of knowledge Misconceptions and Stigma Confidentiality of the information Awareness of the importance of examination The Cultural differences in society affecting concepts of prevention and early detection	
Capacity Building in Human Resources and Logistic Support	Lack of competencies Inadequate distribution of human resources Lack of logistical resources and modern equipment	Increasing the budget allocation for early detection programs Increasing the logistical and human resources to increase the coverage rate by training and manpower Linking the annual performance of general practitioners in primary healthcare centers Hosting independent international experts regularly to evaluate the program and staff Raising the level of efficiency of employees through training courses

**Table 5:** Vision, objectives, and strategy of the UAE cancer control plan.

Vision	To reduce deaths through multiple interventions including early detection of cancer in the UAE
Message	We fight cancer in the UAE hand in hand with our partners and work with innovation and exercising best practices. We are embarking to assemble comprehensive competencies and human resources for a disease-free UAE society
Objectives of the Strategy	1. To strengthen the planning and implement it at the national level to combat cancer in the United Arab Emirates 2. To reduce premature and preventable deaths and the risk of developing cancer by 25% by the year 2025 3. To strengthen cancer prevention, early detection, and treatment 4. To ensure sustainability and continuous development of cancer prevention, control, and treatment strategies 5. To enhance the quality of life for cancer patients 6. To ensure continuum of care through well-defined transition points in the healthcare system 7. To develop a framework to enhance, integrate, and coordinate initiatives to combat cancer and outline principles and regulations to supervise the organization 8. To ensure consistency and standardization in practices and help unify efforts in the fight against cancer 9. To consolidate the efforts by providing a legal framework of applicable governmental regulations and policies

**Table 6:** Recommendations of WHO and EMRO for cancer control plans.

1.	Governance: Focus on developing a strategy and a multi-sectoral committee for the prevention and control of cancer; while ensuring the availability of a sustained budget, adequate national cancer rates, setting costs for care and treatment packages, and determining a mechanism to cover treatment expenses with equity
2.	Prevention: Focus on adopting healthy life by combating smoking, encouraging physical activity, and healthy food in line with the Non-Communicable Disease control framework/plan. The focus should include vaccinations against hepatitis and HPV
3.	Early detection: The strategic directions in this area are based on four axes: Awareness of the population about early warning signs and symptoms of cancer, education, continuing education for health staff on the early signs and symptoms of the common cancers, prompt diagnosis and referral for patients, screening programs, and a continuous evaluation of the effectiveness of these programs. The focus should also be employed on accessibility and the affordability of diagnostic tests for suspected cases.
4.	Treatment: Focus on developing and implementing protocols and best clinical practices based on evidence-based guidelines. Assess human resource needs and focus on accessibility and affordability of cancer care services and extend affordable treatment packages. This must also include the development of integrated, coordinated and prompt referral systems to avoid delays in diagnosis and treatment.
5.	Palliative care: There is a need to develop integrated multidisciplinary palliative care services including pain care and psychological support, available in hospitals and primary healthcare centers. Developing and implementing protocols for best evidence based clinical practice and integrated care, and a swift and early transition. Palliative care should be included in medical academic programs.
6.	Research and Surveillance: Develop a population based national cancer registry, hospital registries, and monitor these with an accredited quality assurance program. The area includes focusing on developing and implementing an integrated plan for research according to the priorities of the country.

**Table 7:** Strategic axes of the UAE cancer control plan.

First Area
1. Education and understanding
Enhancing health awareness about the knowledge of cancer, risk factors leading to cancer and correction of misconceptions about the disease
2. Prevention
Launching awareness campaigns and prevention programs against cancer and known causes
3. Early detection
Detecting cancer in the early stages to increase the patients’ survival and outcome. It involves periodic clinical assessments and reduces the delays in appropriate treatment referrals to receive treatment promptly
4. Rapid diagnosis
A healthcare center should assess condition of the patient promptly in a systematic integrated way and take appropriate medical decisions based on a developed pathway
**Second Area**
5. Treatment
Provide appropriately validated clinical practices in line with international guidelines for treating cancer according to disease stage to improve outcomes
6. Ongoing care
Provide timely transition to post-treatment and palliative care services for cancer patients and educating them about the appropriate ways to live with the disease and directions to avoid disease recurrence
**Third Area**
7. Performance measurements
Establishing national records including all data sources in a central place and assemble comprehensive data of high quality, accuracy, and to record information about a disease
8. Workforce capacity building
Providing a qualified and appropriately trained team to deliver prevention, treatment, continuity of care for patients; and provision of suitable training facilities for the workforce
9. Research
Cancer research improves the diagnosis, treatment, outcome, and enhance quality of life by translating quality research and clinical trials for improvements in personalized care

**Table 8:** Detailed strategic and executive framework of the UAE cancer control plan.

Strategic Axis	I. Education and Understanding			
Main objectives	Application mechanisms	Measurement indicators	The executing agency	Follow-up
Raising health awareness about cancer and associated risk factors and correcting the misconceptions	Conducting a national survey on awareness in society assessing knowledge of risk factors and opinions about access to services and early examination	Survey completion rate	Ministry of Health and Protection (Department of Specialized Care)	Ministry of Health and Protection (Department of Specialized Care)
	Raising health awareness about cancer risk factors Inclusion of cancer in scientific curricula The initiative of the researcher/young intellectual which aims and implements the cancer awareness campaigns in school and university	Number of awareness campaigns	Stakeholders	
	Awareness campaigns synchronized with designated international days for each cancer type	Number of awareness campaigns	Stakeholders	
**Strategic Axis**	**Ⅱ. Prevention**			
Main objectives	Application mechanisms	Measurement indicators	The executing agency	Follow-up
Monitoring of risk factors between different groups in society and encouragement to adopt healthy lifestyles	Physical activity Inclusion and intensification of physical activity compulsory in schools Campaigns to encourage exercise and walk in the community Creation of more tracks for walking and parks within reach of people	The number of awareness programs	Ministry of Health and Prevention /Care department Specialization/Promotion of health management in participating parties	Ministry of Health and Prevention
	Healthy foods Develop educational programs on healthy diet	The number of awareness programs		
	Assessment of the presence of carcinogenic factors in the environment and highlighting the environmental pollution and exposure to radiation	The number of awareness programs		
	Awareness campaigns about the harms of smoking and shisha in young people	The number of awareness programs Monitor smoking rates		
Providing preventive vaccinations	Hepatitis B vaccination for prevention of liver cancer for high-risk population	Hepatitis C vaccination coverage rate Children and among those who have major risk	Ministry of Health and Protection/All health authorities	
	HPV vaccination in schools and society for girls aged 13–26 years to prevent cervical cancer	Coverage rate of the targeted category		
**Strategic Axis**	**Ⅲ. Early Detection**			
Main objectives	Application mechanisms	Measurement indicators	The executing agency	Follow-up
Create a national program for early detection of cancer	Development of a central public electronic recall system for early detection services and identification by e-mail	Completion rate Population coverage rate in target groups	Ministry of Health and Prevention Society/Childhood Department Maternity/Statistics Department and research	Ministry of Health and Prevention the society
	Create a national platform or program for registration of cases that underwent early examination for cancer			
	Increase the capacity of logistical and human resources to increase population coverage			
Health insurance and financial coverage for early disclosure	Insurance coverage for early diagnostic examinations Discounted packages for early detection and uniform prices in the private and public sector	Completion rate		
Increase awareness about the importance of early detection	Awareness campaigns on the importance of early screening Facilitate visitors to healthcare centers Target age and annual performance linked for health workers in early examination centers The rate of turnout to early examination. Appointing clinical nurse specialists to educate and support the team	Number of awareness campaign Percentage of cases from categories of target age transferred for early examination Number of specialized workforces that was redundant	Ministry of Health and Prevention (Community/Participants)	Ministry of Health and Prevention
Establish a framework and governance policy for quality assurance and early screening services in the health regions	Develop a framework for standardization and best of practices Clinical pathways for early cancer examination For early screening of breast, cervical, and colon cancer among the target age groups in the population	Percentage of policy completion and frameworks	Ministry of Health and Prevention community/care management Specialty	
Raising awareness of common symptoms of cancer in society	Awareness campaigns about the symptoms of the most common cancers in the community	Number of awareness campaigns	All participating parties	
**Strategic Axis**	**Ⅳ. Rapid Diagnosis**			
Main objectives	Application mechanisms	Measurement indicators	The executing agency	Follow-up
Establish an effective referral system between different levels of care for cancer patients	Implementing the Service Access Policy so that access time for diagnostic and therapeutic services is reduced every year	2. Average waiting time from the time of onset to GP referred to the specialist 3. Average waiting time from doctor’s appointment till the diagnosis 4. Average waiting time from diagnosis time until start of the treatment 5. Average waiting time from GP appointment to time received treatment	Service providers, Early examination and Therapeutic services	Ministry of Health and Prevention Participating parties
Rapid lung cancer diagnosis initiative	Launch of rapid mobile investigation clinics for early detection of lung cancer using X-ray, CT scan, and breath examination for people susceptible to lung cancer	Number of beneficiaries	Entities involved in cooperation With AstraZeneca	
**Strategic Axis**	**Ⅴ. Treatment**			
Main objectives	Application mechanisms	Measurement indicators	The executing agency	Follow-up
Covering the cost of cancer treatment	Adopt a Pay for performance model (Personalized reimbursement model). Dubai health authority is providing therapeutic services where the treatment is covered by insurance and pharmaceutical companies. The results are then evaluated on treatment response/efficacy	Number of beneficiaries	Healthcare service providers	Ministry of Health and Prevention Participating parties
Accreditation of centers of excellence for cancer treatment	Preparing for specialized centers of excellence (third level) in cancer treatment and its complications and rehabilitation centers	Number of accredited centers of excellence	Stakeholders	
**Strategic Axis**	**Ⅵ. Ongoing Care**			
Main objectives	Application mechanisms	Measurement indicators	The executing agency	Follow-up
Development of palliative care services	Studying the work on adding the palliative service in health centers with easy access of services and develop a guide for implementing a palliative care program	Percentage of completion of the guide	Ministry of Health and Prevention Stakeholders	Ministry of Health and Prevention Participating parties
	Creation of teams to support cancer patients. The team consists of patients who have recovered or are under-treatment The team meets periodically for psychology support among patients	Team achievement percentage Number of beneficiaries	Stakeholders	
**Strategic Axis**	**Ⅶ** **. Performance Measurements**			
Main objectives	Application mechanisms	Measurement indicators	The executing agency	Follow-up
Annual evaluation for anti-cancer performance indicators	Establishing a registry for early detection of cancer Preparing and developing a registry for early detection and development Early detection data collection	The percentage of completion of the national registry, population coverage for early cancer screening among the targeted age groups	Ministry of Health and Prevention Department of Research and Statistics Participating parties	Ministry of Health and Prevention Department of Research and Statistics
	Create a unified national electronic registry for cancer	The percentage of completion of the national cancer registry	Ministry of Health and Prevention Society/Statistics Department of Research and Statistics Participating parties	Ministry of Health and Prevention Department of Research and Statistics
	Measuring indications: The rate of detection of positive cases by early cancer examination Average number of cases detected in late stages of cancer Waiting period, since the case was referred before to the general practitioner to complete the early examinations	Report completion percentage rate of time commitment to access the service	Ministry of Health and Prevention Participating parties	Ministry of Health and Prevention community/care management Specialty
	Measurement of the KAP index (knowledge, attitude, and practice-to assess acceptance) for the community’s understanding of cancer screening	Report completion rate	Ministry of Health and Prevention Participating parties	Ministry of Health and Prevention community/care management Specialty
**Strategic Axis**	**Ⅷ** **. Research**			
Main objectives	Application mechanisms	Measurement indicators	The executing agency	Follow-up
The priority for epidemiological and clinical research of cancer	Research work to discover concepts, knowledge and opinions about cancer, risk factors, and screening cancer in the context of encouraging research related to cancer	Research completion rate	Ministry of Health and Prevention Participating parties	Ministry of Health and Prevention community/care management Specialization/Management Statistics and research
	Develop a research agenda for the three most common cancers (breast, colon, and thyroid)	Agenda completion rate	Ministry of Health and Prevention Participating parties	
**Strategic Axis**	**Ⅸ** **. Workforce**			
Main objectives	Application mechanisms	Measurement indicators	The executing agency	Follow-up
Providing qualified human resources in the developing field of cancer	Complete medical team specialized in treatingcancer in secondary and specialty care Specialized doctors Clinical nurse specialist X-ray technicians	The percentage of increase in workforce	Ministry of Health and Prevention Participating parties	Ministry of Health and Prevention community/care management Specialty
Raising the efficiency of employees, healthcare professionals	Creation of training programs for healthcare workers in the field of cancer and assess the risk factors such as: Awareness and health education Early detection Palliative care	The number of training programs	Ministry of Health and Prevention Community/Training Center and development Participating parties	

## Abbreviations

EMRO: Eastern Mediterranean Region; ICER: Institute for Clinical and Economic review; KPI: key performance indicators; MENA: Middle East and North Africa; MDT: multi-disciplinary team; NICE: National Institute for Health and Care Excellence; NCD: non-communicable diseases; UAE: United Arab Emirates; WHO: World Health Organization.
